# Local comparison of protein structures highlights cases of convergent evolution in analogous functional sites

**DOI:** 10.1186/1471-2105-8-S1-S24

**Published:** 2007-03-08

**Authors:** Gabriele Ausiello, Daniele Peluso, Allegra Via, Manuela Helmer-Citterich

**Affiliations:** 1 Centre for Molecular Bioinformatics, Department of Biology, University of Rome "Tor Vergata", Rome, Italy

## Abstract

**Background:**

We performed an exhaustive search for local structural similarities in an ensemble of non-redundant protein functional sites. With the purpose of finding new examples of convergent evolution, we selected only those matching sites composed of structural regions whose residue order is inverted in the relative protein sequences.

**Results:**

A novel case of local analogy was detected between members of the ABC transporter and of the HprK/P families in their ATP binding site. This case cannot be derived by events of circular permutation since the residues of one of the region pairs are located in reverse order in the sequence of the two protein families. One of the analogous binding sites, the one identified in HprK/P, is known to also bind pyrophosphate, which is used as preferred energy source in its kinase and phosphorylase activity.

**Conclusion:**

The discovery of this striking molecular similarity, also associated to a functional similarity, may help in suggesting new experiments aimed at a deeper understanding of members of the ABC transporter family known to be involved in many serious human diseases.

## Background

The global comparison of protein sequences or structures is one of the most used computational tools in the analysis of newly discovered proteins [1]. These methods can highlight evolutionary relationships. However, due to possible events of divergent evolution in the functional site/s, they cannot always allow the "guilt by association" inference of a protein function.

The vast majority of known functions (enzymatic activities, binding sites etc.) are encoded by a relatively small set of residues located in a conserved geometry both in the protein sequence and in the protein structure. 3D motifs [2,3] can thus be used, in different forms, for analyzing and inferring molecular functions when the global similarity is not conserved (for a review see [4]).

Local similarities in the context of a global non-similarity, are generated by phenomena of divergent [5] or convergent evolution. The latter are uncommon and few are described in the literature, well-known examples being represented by the SHD catalytic triad in serine proteases [3,6] or by the region surrounding the ploop in many nucleotide-binding proteins [7,8].

Methods for the identification of local structural similarities alone are not sufficient to spot cases of convergent evolution. Here we applied a new approach that consists of an exhaustive search for local structural similarities between known protein structures, followed by a selection of structural similarities coming from different regions and located in a different order in the sequence of the protein families sharing the site.

We analyzed the results of an all-versus-all local comparison in an ensemble of protein functional sites, searching for 3D matches characterized by sequence inversion events. Non-collinear matches which also have a strong statistical significance were manually analyzed and a few cases of convergently evolved sites were identified and are discussed below.

## Results

### Structural comparison experiment

Starting from a non-redundant structural dataset of about 2000 protein chains, we identified 10175 surface cavities. About 2500 of those cavities were defined as functional since they contain a consistent fraction of residues associated to a PROSITE [9] pattern or a known ligand binding site (see Methods). We performed an all versus all structural comparison of the functional clefts with the whole dataset of 10000 clefts in search of significant structural similarities.

For the cavities comparison, the sequence-independent Query3D algorithm [10] was used. We set highly stringent parameters to detect only strong similarities and selected only 3D matches comprising of at least 50% of known functional residues (see Methods). We obtained a total of about 66000 structural matches among protein cavities ranging from 2 to 10 residues in size. In Table 1 the distribution is reported of the number of matches with respect to their length.

### Selection of non collinear and significant matches

We selected only those matches whose matching residues are non-collinear in the corresponding protein sequences. To do so, the list of matches has been searched for non-collinearity between the paired residues, see Methods. Table 1 reports the number of matches that are collinear and the matches that are not collinear, for each match length.

We calculated a significance value for each one of these matches in the form of a Z-score. Only matches longer than 7 residues and with a Z-score higher than 10 were analyzed. This threshold is considerably stringent, as can be deduced from different tests performed in other massive structural comparison experiments [11]. This stringency in statistical significance strongly supports the hypothesis that only real structural similarities have been considered.

### Analysis of non collinear matches

A total of 32 non-collinear structural matches were selected within these stringent thresholds and manually analyzed (see Table 1). 28 matches were identified as common cases of non-collinearity deriving from events of circular permutations of protein sequences [12].

**Figure 1:**
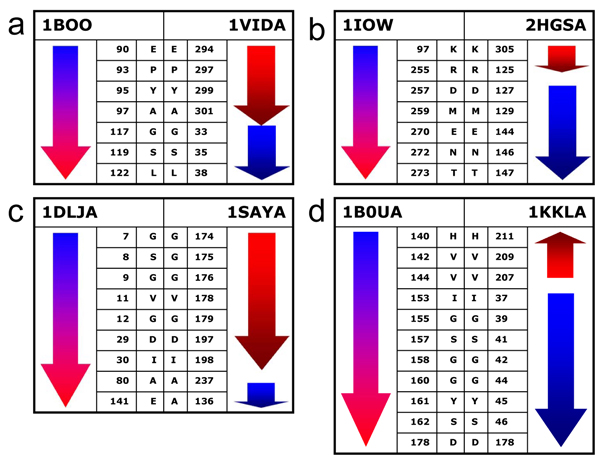
**Details of structural matches**. PDB codes and chain identifiers are reported for each one of the selected structural matches together with the number and type of aligned amino acids. The arrows describe permutations and inversions in protein sequences; the N to C-term direction is colour-coded (blue is associated to N-term and red to C-term). a) The residues involved in the match bind S-adenosyl-homocysteine (1boo) and S-adenosyl-methionine (1vidA), and share a high structural similarity. b) Matching residues bind ADP, Mg and PHY in the 1iow structure; and ADP, Mg and GSH in the 2hgs structure c) The 1dljA residues involved in the match bind a 1,4-dihydronicotinamide adenine dinucleotide; 1say is 92% identical to 1pjc, which binds nicotinamide-adenine-dinucleotide with the residues involved in the 3D match. d) In 1b0uA, the matching residues bind an ATP molecule; 1kklA is 100% identical to 1jb1A, which binds PO4 with the residues involved in the 3D match (see Results).

Of the remaining 4 cases, three are already known in the literature and involve: the N-4 Cytosine-Specific Methyltransferase Pvu II and Catechol O-Methyltransferase [13] (Z-score 11.61); Udp-Glucose Dehydrogenase and L-Alanine Dehydrogenase [14] (Z-score 13.55); D-Ala Ligase and human Glutathione Synthetase [15] (Z-score 14.28). For details on these matches see Figure 1(a-b-c).

### A new case of sequence inversion

The fourth case (z-score 12.14), see Figure 1d, involves one member of the ABC transporters family and a bacterial Hpr kinase/phosphorylase protein (PDB codes: 1b0u and 1kkl, respectively). This 3D similarity belongs to the category of inverted structural matches (see Method) and cannot have arisen from simple events of sequence rearrangements, therefore identifying a true and almost unique case of convergent evolution.

The ATP binding subunit of a bacterial histidine permease belongs to the ABC transporters family [16], whose members are widespread into different phyla, from bacteria to humans. Some of the ABC transporters are known to be involved in several human disorders, such as cystic fibrosis, muscular dystrophy, adrenoleukodystrophy, Stargardt disease and others. The bacterial HPrK/P [17], on the other hand, is a bacterial sensor enzyme that plays a major role in the regulation of carbon metabolism and sugar transport, controlling the expression of numerous catabolic genes; it catalyzes the ATP- as well as the pyrophosphate-dependent phosphorylation of Ser-46 in HPr, a phosphocarrier protein of the phosphoenolpyruvate-dependent sugar phosphotransferase system (PTS).

**Figure 2:**
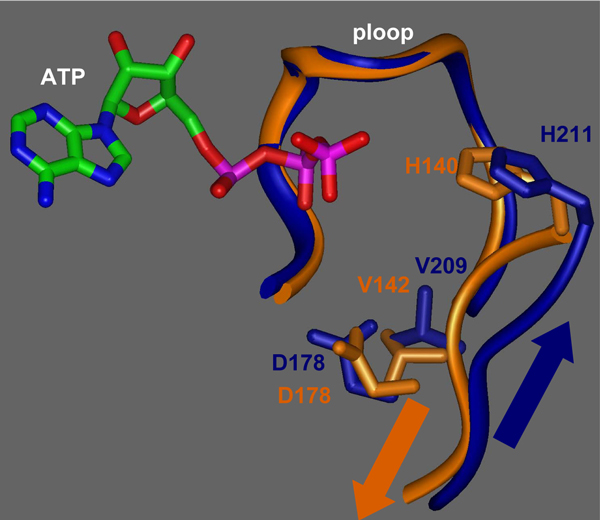
**Conserved residues in the identified 3D match**. Graphical view of the conserved residues in the identified 3D match. The ABC transporter (PDB code: 1b0uA) is shown in blue and the HPrK/P (PDB chain code: 1kklA) is in orange. The ploop of the two proteins is represented as a ribbon. The two beta filaments where the catalytic histidine and the hydrophobic residues are encoded in opposite order are also shown as ribbons. The ATP molecule co-crystallized with the histidine transporter subunit is shown colored by atom type; some of the side-chains involved in the functional match are also shown and are identified by their PDB residue number. The two arrows indicate the N to C orientations of the oppositely oriented beta filaments.

**Figure 3:**
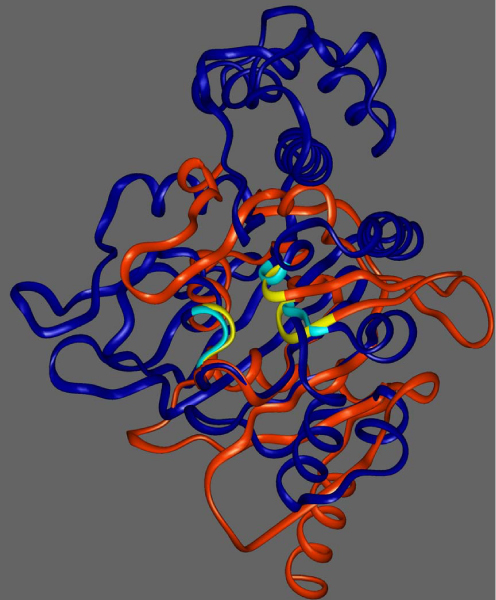
**Superimposition corresponding to the local 3D match**. The structural superimposition corresponding to the local 3D match described in Figure 2. The ABC transporter and the HPr K/P protein chains are shown as blue and red ribbons. The local match of 1b0uA and 1kklA residues is highlighted in cyan and yellow, respectively.

**Figure 4:**
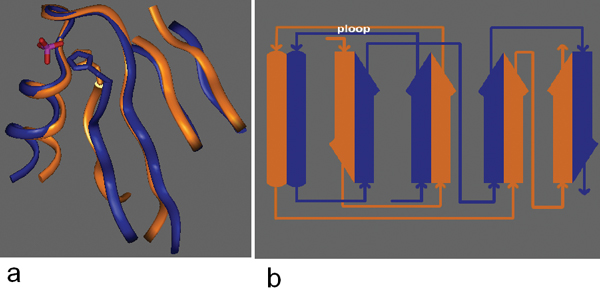
**Superposed secondary structure elements**. a) Ribbon representation of the conserved SSEs surrounding the ATP/PP binding site: four beta filaments and an alpha-helix. The 1b0uA chain is shown in blue, the 1kklA chain in orange. The four beta filaments form a beta-sheet, whose second filament corresponds in both structures to the ploop preceding sequence. The helix corresponds to the sequence immediately following the ploop structure. The superposition of the two beta-sheets shows the two internal filaments displaying the same orientation in space (in both structures, the filaments point up in an N to C direction). The two external filaments point in opposite directions in the two structures. The first filament on the left side of the picture corresponds to the sequence surrounding the catalytic histidine; the fourth filament on the right is not involved in any known specific functional region. b) Topology of the superposition. A simplified view of the superposed structure is shown with the same colors used in Figure 4a. Beta filaments are represented as arrows, pointing from N to C; the helices are represented as cylinders. The protein stretches joining the secondary structure elements are depicted with no relation to their real length.

The nucleotide binding site of the ATP-binding subunit of the histidine permease from Salmonella typhimurium and the phosphate binding active site of the Hpr kinase/phosphorylase from Lactobacillus casei are built through the juxtaposition of three distinct residue stretches (see also Table 2): i) the ploop; ii) a pair of negatively charged residues (D178-E179 in 1b0u; D178-D179 in 1kkl) and iii) a catalytic histidine followed (in the case of Hpr K/P) or preceded (in the case of the ABC transporter) by three hydrophobic residues on a beta filament (Figure 2). The third stretch proposes the residue side-chains in very similar positions in 3D, but encoded in opposite order in the protein sequence (N to C terminus in one protein and C to N terminus in the other). The two structures look quite different after superposing the conserved residues (Figure 3), but they share a very similar core composed of 4 beta filaments and one helix (Figure 4a). One of the filaments and the helix respectively precede and follow the ploop structure; two of the three remaining beta strands follow the same direction but are oppositely oriented (Figure 4b). This finding can support the hypothesis that the structural context is necessary for the stability and function of the described ATP/PP binding site, even if some of the participating SSEs share opposite directions.

In both proteins, the residues identified belong to a well-studied functional site, described in detail below. The co-crystallized molecules (an ATP and a pyro-phosphate) neatly superpose the corresponding phosphate atoms, in support of a functional meaning of the 3D similarity. This quite unique situation accounts for the fact that this striking similarity has not been highlighted so far.

A multiple alignment of members of the two protein families derived from the corresponding superposition of the functional sites is shown in Table 2.

## Conclusion

An exhaustive search has been performed for significant 3D similarities between protein functional sites containing residues that are non-collinear in the respective protein sequences. From a non-redundant set of protein structures, an ensemble of about 10,000 cavities were defined, one fourth of which could be associated to a known molecular function using PROSITE patterns and/or bound ligands.

The local comparison produced more than 60 thousand 3D matches. In this list, a relatively low number of matches appeared to involve non-collinear residues. All matches were evaluated for their statistical significance using the Z-score. Cases with Z-score > 10 were carefully analyzed and manually inspected in the graphics.

Four interesting cases were identified, three of which were already known in the literature as cases involving a permutation in one of the protein families comprised in the structural match.

The fourth case involved a member of the ABC transporter family and a bacterial HprK/P (Figures 2 and 3). Three regions in the two protein sequences are involved in the structural match: their location in the respective sequences is 1-2-3 versus 3-1-2. This is compatible with a circular permutation in one of the protein families. But in one of the regions (the one identified by #3 in the preceding sentence) the residues are located in inverse order in the two sequences, therefore suggesting that a case of convergent evolution has occurred.

Interestingly, a structural core composed of 4 beta filaments and one helix is conserved in the two structures (Figure 4), but two of the beta filaments are oriented in opposite directions. This finding can be relevant both for a better understanding of structure-function relationships and for medical significance, since members of the ABC transporter family are involved in several human diseases, such as cystic fibrosis, muscular distrophy, adrenoleukodystrophy, Stargardt disease and others. This analogy might help in suggesting experimental strategies to devise new classes of inhibitors, peptides or compounds.

## Methods

### Cavities dataset

We used a NCBI non-redundant PDB [18] composed of 1924 chains obtained using only X-ray solved structures and a sequence-similarity cut-off corresponding to a minimum BLAST p-value of 10e-7.

Using the SURFNET algorithm [19] we identified a dataset of 10175 surface clefts on these chains with a cavity volume higher then 200 Å^3^.

We defined each cleft as the set of residues identified by the algorithm that surrounds the cavity pocket [20].

### Functional residues

Functionally important residues were identified in the set of defined cavities by searching for PROSITE [9] patterns and ligand binding sites.

PROSITE motifs were searched in the sequences of our protein dataset with the ScanProsite algorithm [21]. All PROSITE regular expressions were used excluding those marked as "unspecific".

Ligand binding residues were identified with a distance criterion. All residues within 3.5 Å distance from any HETEROATOM found in the selected co-ordinate set were selected and assigned to the category.

All those clefts displaying less than 75% of the residues identified to be involved in one of the defined functions were discarded, with the purpose of considering only almost complete functional sites [20].

### All vs. all structural comparison

The structural comparison was performed using the sequence independent local comparison algorithm Query3D [10]. Matching criteria of Query3D are both geometrical (r.m.s.d. between paired residues) and biochemical (scores from a substitution matrix). The algorithm uses a two-point representation of each residue, the C-alpha and a side-chain representative point. An exhaustive exploration guarantees finding the two largest sets of matching aminoacids in a pair of protein structures. For this experiment, high stringency parameters were set (r.m.s.d. < 0.7 Å and residue similarity > 1.2 according to the Dayhoff substitution matrix) in order to obtain only matches with a high similarity. Whenever a match involves more than 10 aminoacids, only the first ten are considered.

In the all vs. all comparison experiment, all surface clefts containing at least 75% of functionally important residues were compared to the whole set of clefts. Moreover, the algorithm was forced to consider only the structural similarities involving at least 50% of aminoacids being annotated as functionally important [20]. This requirement helps in selecting only matches in protein regions characterized by an easily deducible function.

### Matches significance

Each match is scored with the match length, i.e. with the number of residues that can be superposed within the defined similarity thresholds. The significance of each match is evaluated by calculating the Z-score over the value distribution of the query cleft comparison with the whole dataset. For each match, the Z-score is computed as the difference between the value of the match and the average value of all the matches for the query patch, divided by the standard deviation.

### Definition of collinear and inverted structural matches

A structural match can be described as a set of pairs of residues that can be superposed in 3D. Each residue pair is identified by an uppercase letter (i.e. A) and the two composing residues with the same letter in lowercase followed by one or two apice depending on its belonging to the first or the second structure (i.e. a', a").

Given two residues a' ∈ A and b' ∈ B, a' < b' if a' precedes b' in the primary sequence. Two pairs A and B are non-collinear if a' < b' while b" < a" or if b' < a' while a" < b".

A structural match is non-collinear if it contains at least 2 non-collinear pairs, and is inverted if it contains at least 3 pairs, each of these non-collinear between each other.

## Authors' contributions

GA conceived the study, carried out the structural comparisons and drafted the manuscript. DP carried out the analysis of non-collinear matches. AV participated in the design of the work. MHC participated in the design of the work and in its coordination. All authors read and approved the final manuscript.

## Figures and Tables

**Table 1:** Number of structural matches found. Number of structural matches found between functionally annotated and whole ensemble of surface cavities.

matches	total	2	3	4	5	6	7	8	9	10
collinear	33966	4893	6109	17905	2283	1524	376	486	105	285
non-collinear	31944	3920	8230	18998	621	122	14(7)	23(12)	4(4)	12(12)
total	65910	8813	14339	36903	2904	1646	390	509	109	297

The first and second row reports the number of collinear and non collinear matches, respectively, per each match length. In the third row the total number of matches is detailed. The number in parentheses indicates non-collinear matches with Z-score higher than 10.

**Table 2:** Local multiple alignment of the two families.

Type	PDB code	SWISSPROT code	Residues in inverted regions					Residues in ploop	Negative charges		Ligand	Organism	Decription
Type 1	1b0uA	Q5PN38_SALPA	H211	T210	V209	V208	V207	39–46	D178	E179	ATP	*Salmonella typhimurium*	ATP-Binding Subunit Of The Histidine Permease
	1l2tA	Y796_METJA	H204	T203	V202	V201	V200	38–45	D170	E171Q	ATP	*Methanococcus jannaschii*	Hypothetical ABC Transporter ATP-Binding Protein Mj0796
	1q12A	MALK_ECO57	H192	T191	V190	Y189	I188	36–43	D158	E159	ATP	*Escherichia coli K12*	ATP-Bound E. Coli Malk, Maltose/Maltodextrin Transport
	1g291	Q9HH32_PYRFU	H198	T197	V196	Y195	I194	36–43	D164	E165	POP	*Thermococcus litoralis*	Maltose Transport Protein Malk
	1e3mA	Q8VVV1_ECOLI	H728	T727	A726	F725	L724	614–621	D693	E694	ADP	*Escherichia coli*	DNA Mismatch Repair Protein Muts
	1ewqA	MUTS_THEAQ	H696	T695	A694	F693	L692	583–590	D662	E663	-	*Thermus aquaticus*	DNA Mismatch Repair Protein Muts
	1fnnA	Q8ZYK1_PYRAE	H166	G165	V164	I163	V162	50–57	D131	D132	ADP	*Pyrobaculum aerophilum*	Cdc6P, Cell Division Control Protein 6
		ABCD1_HUMAN	H659	T658	I657	S656	L655	507–514	D629	E630		*Homo sapiens*	adrenoleukodystrophy protein
		CFTR_HUMAN	H1402	E1401	C1400	L1399	I1398	1244–1251	D1370	E1371		*Homo sapiens*	CFTR NBD2

ABC Type 2	1w1wA		L1190	S1189	I1188	V1187	I1186	33–40	D1157	E1158	ATG	*Saccharomyces cerevisiae*	Structural Maintenance Of Chromosome 1, Head Domain Residues 1–214, 1024–1225
	1jj7A	Q96CP4_HUMAN	Q701	T700	I699	L698	L697	538–545	D667	D668	ADP	*Homo sapiens*	Peptide Transporter Tap1, C-Terminal ABC ATPase Domain
	1ji0A	Q9X0M3_THEMA	Q196	E195	V194	L193	L192	38–45	D163	E164	ATP	*Thermotoga maritima*	ABC Transporter
	1xmiA	Q99989_HUMAN	S605	T604	V603	L602	I601	458–465	D572	S573	ATP	*Homo sapiens*	Cystic Fibrosis Transmembrane Conductance Regulator, Nucleotide Binding Domain One
	1r0wA	CFTR_HUMAN	S605	T604	V603	L602	I601	458–465	D572	S573	-	*Mus musculus*	Cystic Fibrosis Transmembrane Conductance Regulator, NDB1 Domain (Residues 389–673)

HPrK/P	1jb1A	HPRK_LACCA	H140	G141	V142	L143	V144	155–162	D178	D179	PO4	*Lactobacillus casei*	Hprk/P Bound To Phosphate, Hprk Protein
	1knxA	HPRK_MYCPN	H139	G140	V141	L142	L143	154–161	D177	D178	-	*Mycoplasma pneumoniae*	Hpr Kinase/Phosphatase
	1ko7A	HPRK_STAXY	H136	G137	V138	L139	V140	151–158	D174	D175	PO4	*Staphylococcus xylosus*	Hpr Kinase/Phosphatase
	1kklA	HPRK_LACCA	H140	G141	V142	L143	V144	155–162	D178	D179		*Lactobacillus casei*	Hprk/P In Complex With B. Subtilis Hpr, Phosphocarrier Protein Hpr
	1kkm	HPRK_LACCA	H140	G141	V142	L143	V144	155–162	D178	D179	PO4	*Lactobacillus casei*	Hprk/P In Complex With *B. Subtilis *P-Ser-Hpr

A sequence alignment of different members of the ABC transporters and HPr K/P families is reported. Column 1 identifies the protein family. ABC transporters are grouped in two different types: a) type 1 members comprise the histidine permease of *S. typhimurium *and other Nucleotide Binding Domains (NBD) of the same family. They all display the same conserved residues, including the catalytic histidine; b) type 2 members display a non-histidine aligned to the histidine of the first group. In many cases, two different nucleotide binding domains (a non-histidine and a histidine) known as NBD1 and NBD2 belong to the same protein sequence, with different functions: NBD1 (without catalytic H) usually displays a regulative function, while NBD2 (with H) encodes a catalytic function. The remaining columns for each protein report: the PDB code, the Swiss-Prot code, the structurally aligned residues in the inverted region, the sequence range of the ploop, the two negatively charged structurally aligned residues, the bound ligand, the organism and a short description.
